# Expression and prognostic significance of CBX2 in colorectal cancer: database mining for CBX family members in malignancies and vitro analyses

**DOI:** 10.1186/s12935-021-02106-4

**Published:** 2021-07-28

**Authors:** He Zhou, Yongfu Xiong, Zuoliang Liu, Songlin Hou, Tong Zhou

**Affiliations:** 1 The Second Department of Gastrointestinal Surgery, Affiliated Hospital of North Sichuan Medical College, 63 Wenhua Road, Nanchong, 637000 Sichuan Province China; 2 The First Department of Hepatobiliary Surgery, Affiliated Hospital of North Sichuan Medical College, Nanchong, 637000 Sichuan China; 3 Institute of Hepatobiliary, Pancreatic and Intestinal Disease, North Sichuan Medical College, Nanchong, 637000 Sichuan China

**Keywords:** Chromobox, Colorectal cancer, CBX2, Prognosis, Cell proliferation

## Abstract

**Background:**

The Chromobox (CBX) domain protein family, a core component of polycomb repressive complexes 1, is involved in transcriptional repression, cell differentiation, and program development by binding to methylated histone tails. Each CBX family member plays a distinct role in various biological processes through their own specific chromatin domains, due to differences in conserved sequences of the CBX proteins. It has been demonstrated that colorectal cancer (CRC) is a multiple-step biological evolutionary process, whereas the roles of the CBX family in CRC remain largely unclear.

**Methods:**

In the present study, the expression and prognostic significance of the CBX family in CRC were systematically analyzed through a series of online databases, including Cancer Cell Line Encyclopedia (CCLE), Oncomine, Human Protein Atlas (HPA), and Gene Expression Profiling Interactive Analysis (GEPIA). For in vitro verification, we performed cell cloning, flow cytometry and transwell experiments to verify the proliferation and invasion ability of CRC cells after knocking down CBX2.

**Results:**

Most CBX proteins were found to be highly expressed in CRC, but only the elevated expression of CBX2 could be associated with poor prognosis in patients with CRC. Further examination of the role of CBX2 in CRC was performed through several in vitro experiments. CBX2 was overexpressed in CRC cell lines via the CCLE database and the results were verified by RT-qPCR. Moreover, the knockdown of CBX2 significantly suppressed CRC cell proliferation and invasion. Furthermore, the downregulation of CBX2 was found to promote CRC cell apoptosis.

**Conclusions:**

Based on these findings, CBX2 may function as an oncogene and potential prognostic biomarker. Thus, the association between the abnormal expression of CBX2 and the initiation of CRC deserves further exploration.

## Background

Colorectal cancer (CRC) remains one of the top three causes of tumor-related deaths worldwide [1]. In 2019, an estimated 145,600 new cases of CRC and 51,020 deaths from the disease were reported in the USA [2]. Although the incidence of CRC has decreased in developed countries, due to improved treatments and early screening, the 5-year overall survival (OS) rate of CRC patients in developing countries is still not ideal [3, 4]. The tumor node metastasis (TNM) staging system developed by the American Joint Committee on Cancer and Union for International Cancer Control is the most universal tumor staging standard in the world and an important reference index for predicting the prognosis of tumor patients [5]. Increasing evidence has indicated that CRC is a highly heterogeneous disease with multiple molecular pathways involved in its progression [6]. Nevertheless, current TNM staging systems cannot reflect the intrinsic biological heterogeneity of CRC, especially in patients with atypical early symptoms. This results in < 50% of CRC being diagnosed early, with certain CRC patients diagnosed through this system already presenting with distant metastasis [7]. Therefore, to improve the diagnosis, prognosis and targeted therapy of CRC, it is necessary to find biomarkers that can accurately predict its progression and therapeutic effect, and explore the mechanism of CRC development at the molecular level.

Chromatin has been divided into different domains according to the function of associated genomes, including euchromatin and heterochromatin [8]. The structural distribution and assembly of chromatin are influenced by numerous factors. The proteins that control chromatin dynamics play a pivotal role in the epigenetic regulation of gene expression [9]. The Chromobox (CBX) family proteins are crucial components of chromatin-related complexes heterochromatin protein 1 (HP1) and polycomb (Pc), which are involved in transcriptional regulation, chromatin structural modification, and the cell development process [10]. To date, eight members of the CBX family proteins have been identified in eukaryotic organisms, each containing a single N-terminal chromosomal domain[11]. The CBX family can be subdivided into two groups: One consisting of CBX1, CBX3, and CBX5, with a similar structural feature of HP1 homologs (HP1α, HP1β, and HP1γ), and another made of Pc paralog proteins, known as CBX2, CBX4, CBX6, CBX7, and CBX8, which can recruit Pc repressive complexes 1 to maintain expression patterns of different genes during cell proliferation [12].

CBX family proteins are widely involved in a variety of biological process in all metazoans, including cell cycle control, induction of cell differentiation, and maintenance of pluripotency of embryonic stem cells [13]. Existing evidence has revealed that the dysregulation of CBX proteins results in numerous cell divisions that initiate cancer [14]. For instance, three isoforms of HP1 (CBX1, CBX3, and CBX5) act as organizers of pericentric heterochromatin in conjunction with H3K27me3, which hinders cell cycle progression, leading to transcriptional activation, cell proliferation and cancer [15]. Recent studies have suggested that CBX2 and CBX6 act as oncogenes in hepatocellular carcinoma (HCC). The overexpression of both CBX2 and CBX6 is associated with poor prognosis in HCC patients [16, 17]. Xia et al. [18] demonstrated that mutation of the CBX4 gene causes the transcriptional repression of proto-oncogenes, and can interact with CBX2 and Bmi-1 to alter pre-splicing mRNA. This ultimately causes an abnormal transformation of cells. Unlike other Pc family members, the role of CBX7 as a proto-oncogene or suppressor depends on its specificity in cells and tissues, as well as various epigenetic factors [19].

CBX2 is a key regulator of developmental genes. It shows a stronger effect on cancer progression than other CBX members by repressing the transcription of the Ink4a/Arf locus [20]. Further research has shown that CBX2 is the main protein expressed in ESC and can repress the expression of pluripotency genes that promote stem cell differentiation [20, 21]. Although CBX2 has been reported to be abnormally expressed in a number of cancer types, the role of CBX2 in CRC remains largely unclear. In the present study, integrated analysis of the CBX protein family was performed through several online databases, with the purpose of searching for potential therapeutic biomarkers of CRC patient survival.

## Methods

### Cancer cell line encyclopedia (CCLE) database analysis

CCLE (https://portals.broadinstitute.org/ccle/home) is an open-access database covering large-scale deep sequencing information of 947 human cancer cell lines from > 30 varieties of tissue sources. The mRNA expression of the CBX family in cell lines derived from different tumor types was analyzed by CCLE, to deepen the understanding of DNA mutations, gene expression and chromosome copy number information for specific genes. Gene expression data of the CBX family was downloaded directly from the CCLE website. According to the website, raw microarray data of CRC cell lines were converted to a single value for each probeset using the Robust Multi-array Average algorithm and quantile normalization.

### Oncomine database analysis

Oncomine (http://www.oncomine.org) is a classic oncogene chip database, which integrates data from The Cancer Genome Atlas (TCGA) and Gene Expression Omnibus databases. It provides a variety of analytical tools and visually shows the differences between cancer and normal tissue expression, co-expression analysis, mutation analysis, etc. In the current study, the mRNA expression of distinct CBX family proteins was analyzed between tumor and normal tissues in different types of cancers. The results were filtered using the following threshold: Fold change, 2; P = 1 × 10^− 4^; gene rank, top 10%.

### The human protein atlas database (HPA) analysis

The HPA database (https://www.proteinatlas.org/) provides information on the tissue and cell distribution of all 26,000 human proteins and is publicly available free of charge [22]. This database uses special antibodies and immunohistochemical (IHC) techniques to examine the distribution and expression of each protein in 48 normal human tissues, 20 tumor tissues, 47 cell lines and 12 blood cells. These samples were collected from different clinical individuals, ensuring that the staining results were sufficiently representative. In the present study, IHC images of the CBX protein expression in clinical samples of patients with CRC and normal tissues were obtained from the HPA database.

### Gene expression profiling interactive analysis (GEPIA) database analysis

GEPIA (http://www.gepia.cancer-pku.cn/http://www.gepia.cancer-pku.cn/) is an open-access database for the interactive exploration of multiple cancer genomics datasets. The utilization of GEPIA significantly reduces the barriers in accessing complex genomic data and facilitates rapid, intuitive, high-quality access to molecular profiling and clinical prognostic correlations for large-scale cancer genomics projects. The website query interface is combined with multiple databases that store DNA copy numbers and mRNA expression, gene coexpression, Reverse Phase Protein Array, DNA methylation and clinical survival data. This allows researchers to investigate the interaction between gene alterations and clinical case samples by directly entering gene names. Kaplan–Meier plots were used to compare OS and disease-free survival (DFS) in CRC cases with the mRNA expression of each CBX member.

### Cell culture

The CRC HCT116 and HT29 cell lines, and the normal human colon mucosal epithelial cell line NCM460, were obtained from the Cell bank of the Chinese Academy of Science where they were characterized by mycoplasma detection, DNA –Fingerprinting (Short Tandem Repeat, STR), isozyme detection and cell vitality detection. Cells were cultured in McCoy’s 5 A medium (Gibco, Carlsbad, CA) containing 10% fetal bovine serum (PAN-Biotech, Adenbach, Bavaria) in an incubator with 5% CO_2_ at 37 °C.

### Lentivirus transduction and stable cell line selection

The lentivirus-mediated GV248 vector (Genechem, China) was used to express short hairpin RNA (shRNA) targeting CBX2. The shRNAs sequences targeting CBX2 were as follows:5′- CCGG GAG GTC AAC CCA GGA GAG AGA CTCGAG TCT CTC TCC TGG GTT GAC CTC TTTTTG-3′. The 293T cells were transfected with Lv-shRNA vector for 48 h, the liquid supernatant was collected. Then, HCT116 and HT29 cells (1 × 10^5^ cells/well) were seeded into 24-well plate and cultured in medium for 24 h, then the liquid supernatant was transfected into HCT116 and HT29 cells at a multiplicity of infection (MOI) of 10. The nontarget green fluorescent protein (GFP)-LV vectorwas used as negative control (sh-Ctrl). The stable cell lines were selected using puromycin (4 µg/ml) 72 h after transfection. The medium containing puromycin was replaced every three days for two weeks.

### RNA isolation and RT-qPCR

Total RNA was extracted from cells using RNAiso Plus reagent (Takara, Dalian, China). The Prime Script RT Reagent Kit (Takara) was used to perform reverse transcription following the manufacturer’s protocol. RT-qPCR was performed on a Bio-Rad CFX96 system (Bio-Rad, Hercules, CA). Glyceraldehyde-3-phosphate dehydrogenase (GAPDH) was used as a normalizing control. The primer sequences used in the present study were as follows: CBX2, 5′-GGA ACA TGA GAA GGA GGT GCA G-3′ (forward) and 5′-GAA GAG GAG GAA CTG CTG GAC T-3′ (reverse); GAPDH, 5′-CAG GAG GCA TTG CTG ATG AT-3′ (forward) and 5′-GAA GGC TGG GGC TCA TTT-3′ (reverse).

### Western blotting

Cells were harvested at a density of > 90% and subsequently lysed in RIPA buffer (Beijing Solarbio Science & Technology, China) on ice. Equal amounts of protein lysate (15 µg) were separated using 10% sodium dodecyl sulfate polyacrylamide gels and then transferred onto polyvinylidene fluoride membranes (Millipore, County Cork, Ireland) via electroblotting. Following blocking in rapid block buffer (Sangon Biotech, China) for 15 min, the membranes were incubated at 4 °C overnight with relevant primary antibodies. The Horse Radish Peroxidase-conjugated secondary antibodies (dilution, 1:5000, Cat. No. SE134; Solarbio) were then used to incubate the membranes for 1 h at room temperature. The protein bands were detected by enhanced chemiluminescence (Vilber, Collegien, France). The primary antibodies used were rabbit anti-CBX2 (dilution, 1:2000, Cat. No. ab80044; Abcam), and rabbit anti-GAPDH (dilution, 1:1000, Cat. No. 2118; Cell Signaling Technology).

### Cell colony formation and invasion assays

Colony formation assay was used to test the cell proliferation capacity following CBX2 knockdown. Transfected cells were seeded in six-well plates in triplicate at a density of 500 cells/well and incubated at 37 °C for 2 weeks. The cell colonies were then washed with PBS, fixed with 4% paraformaldehyde for 20 min and stained with 0.2% crystal violet for 30 min. Cell colonies with > 50 cells were identified as positive colonies and the colony numbers were counted under a microscope.

Transwell assay was used to evaluate the invasion ability of colon cancer cells after knocking down CBX2. In brief, 1 × 10^5^ cells were plated in the upper chamber coated of 24-well plate with Matrigel and supplemented with serum-free medium. The lower chamber was filled with culture medium containing 15% FBS. Incubation was carried out for 48 h at 37 °C. The noninvasive cells were scraped off with cotton swabs. The cells that had successfully translocated were fixed with 4% paraformaldehyde, stained with 0.5% crystal violet. The number of invaded cells was observed by using an inverted microscope and calculated by counting six random views.

### Cell apoptosis assay

Flow cytometer (Cytoflex, Beckman Coulter, California, USA) is used to detect cells apoptosis after transfection. Transfected cells were disaggregated using 0.25% trypsin-EDTA solution and washed in PBS twice. Next, 1 × 10^6^ cells were resuspended in PBS and stained with APC-conjugated Annexin V (Cat. No. 88-8007-72, eBioscience, San Diego, California, USA) and 4′,6-diamidino-2-phenylindole (Cat. No. D3571, Invitrogen, Carlsbad, California, USA), according to the manufacturer's recommendations.

### Statistical analysis

The GraphPad Prism (Version 8.0 GraphPad Software, CA) was used for statistical analysis. The significance of differences between groups was evaluated using the Student’s *t*-test. Statistical significance of CBX family expression between CRC and normal tissues from the Oncomine database was provided by the program. Survival data of CBX family mRNA expression were obtained from the GEPIA database. Survival curves were plotted using the Kaplan–Meier method and compared using the log-rank test. P < 0.05 were considered to indicate a statistically significant difference.

## Results

### Dysregulated mRNA expression of CBX family members in CRC

**Fig. 1:**
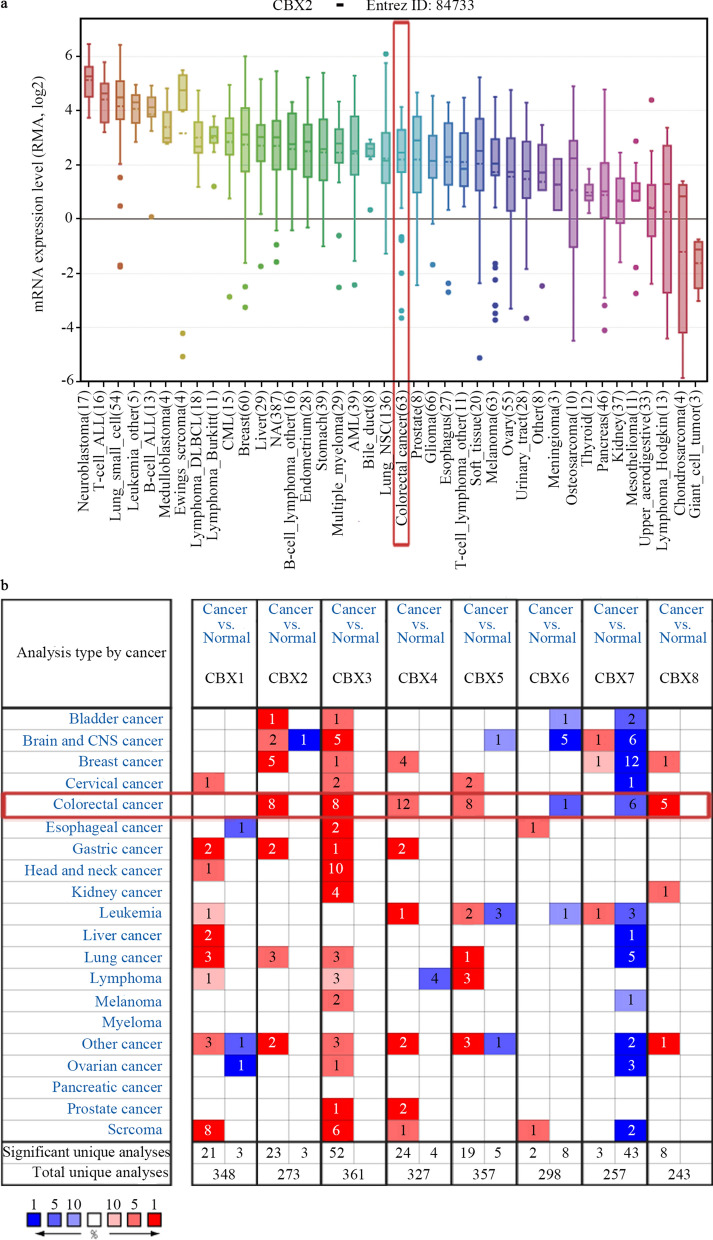
mRNA expression of CBX family members in different types of cancer. **a** mRNA expression level of CBX2 from the CCLE database. The CBX2 mRNA expression level ranked 21th among different human types of cancer (shown in red frame). **b** mRNA expression levels of CBX family members in various types of cancer vs. normal tissues in the Oncomine database. The blue box in the graph indicates that the target gene was lowly expressed in the corresponding tumor, while red indicates highly expressed genes, with statistically significant differences (P = 1 × 10^− 4^). The number in the cell represents the number of studies that meet the set threshold. The color of the cells is determined by the rank of gene expression differences.* CBX* Chromobox,* CCLE* Cancer Cell Line Encyclopedia

The expression of CBX2 in CRC ranked 21th highest among all cancer types, as determined by CCLE analysis (Fig. 1a). Oncomine database analysis showed that the mRNA levels of CBX2, CBX3, CBX4, CBX5, and CBX8 were significantly higher in CRC than in normal tissues, based on a wide variety of datasets. Conversely, CBX6 and CBX7 were confirmed to have a lower expression in CRC, as compared with normal tissue (Fig. 1b).

### Association between CBX mRNA expression and pathological stages of CRC

**Fig. 2:**
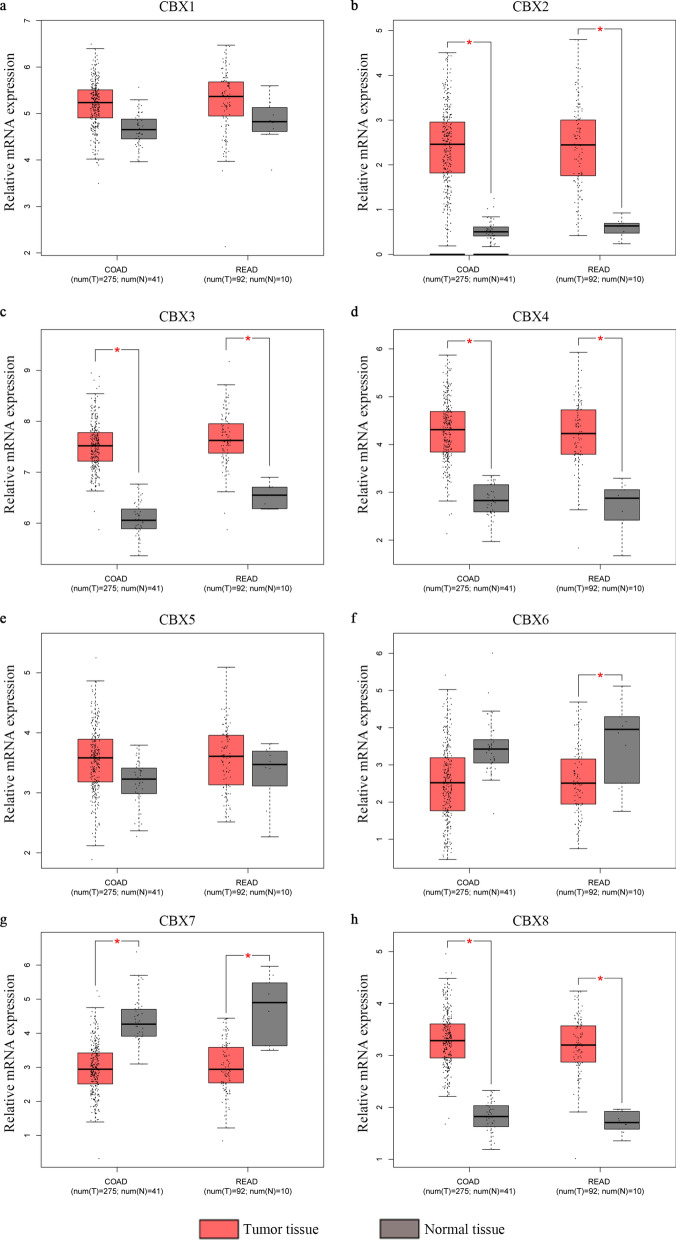
Expression analysis of CBX family members in CRC and normal tissues (GEPIA database). Box plots derived from gene expression data comparing expression levels of a specific CBX family member in CRC and the corresponding normal tissue. Comparison of **a** CBX1, **b** CBX2, **c** CBX3, **d** CBX4, **e** CBX5, **f** CBX6, **g** CBX7, and **h** CBX8 mRNA expression. *P < 0.05. *COAD* colon adenocarcinoma, *READ* rectum adenocarcinoma, *T* tumor, *N* normal, *CBX* Chromobox, *CRC* colorectal cancer, *GEPIA* Gene Expression Profiling Interactive Analysis

**Fig. 3:**
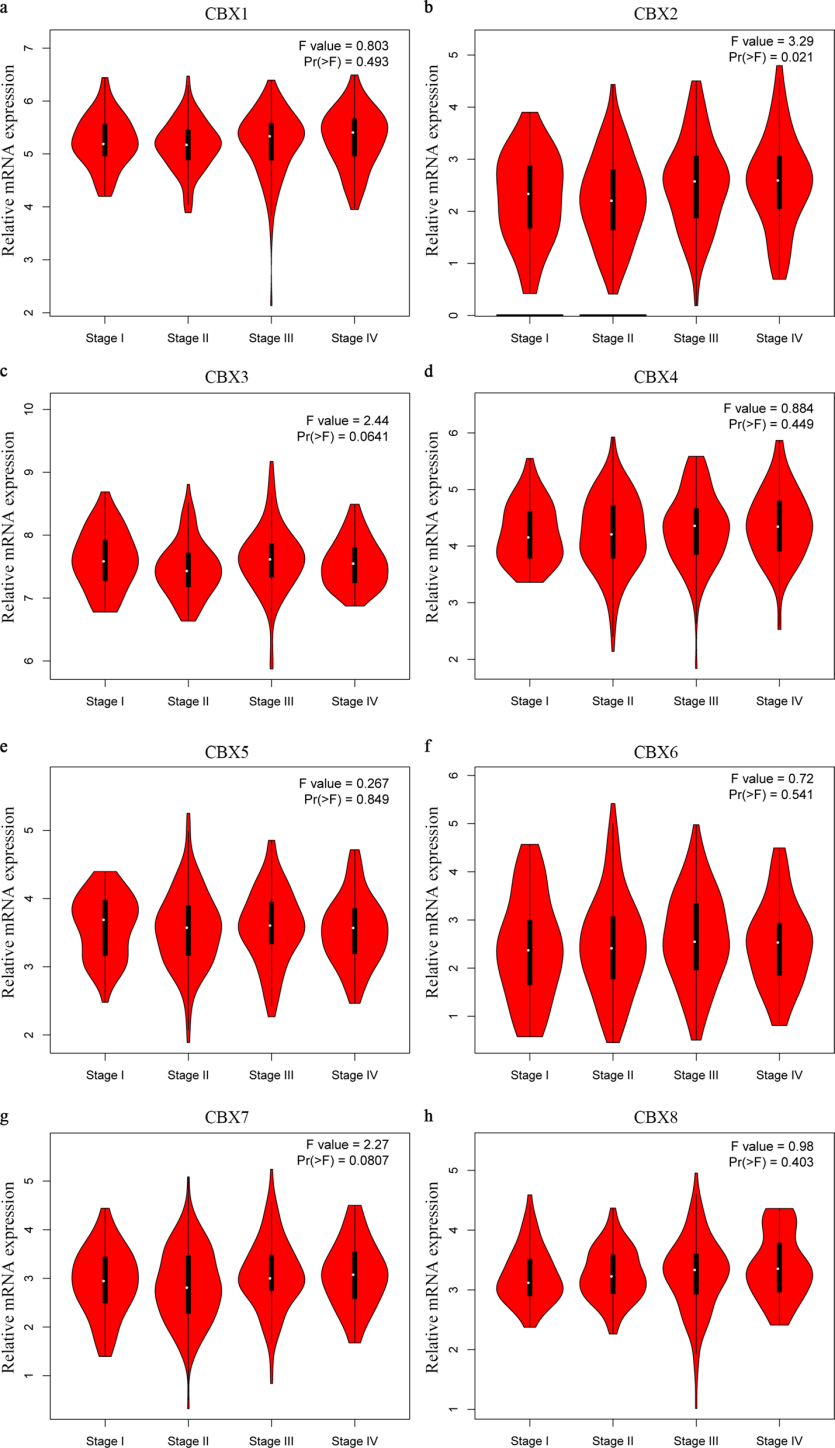
Association between mRNA expression of CBXs and tumor stages in patients with CRC (GEPIA database). **a** CBX1, **b** CBX2, **c** CBX3, **d** CBX4, **e** CBX5, **f** CBX6, **g** CBX7, and **h** CBX8. In the violin plots, the white dots indicate the median; the black box indicates the quartile range; the thin black line indicates 95% confidence interval; the size of the red area indicates the density. F-value, statistical value of the *F* test; Pr (> F), P-value. *CBX* Chromobox, *CRC* colorectal cancer, *GEPIA* Gene Expression Profiling Interactive Analysis

Next, a large sample from the TCGA dataset was analyzed in the GEPIA database. Consistent with the Oncomine database, CBX1-5 and CBX8 transcripts in colon and rectal adenocarcinoma tissues were higher than in normal tissues (Fig. 2a–h). The association between CBX expression and CRC pathological stage was then investigated. Of note, significantly statistical differences between tumor stages I–IV were only identified in the CBX2 group (P = 0.021; Fig. 3b). There was no association between the other CBX members and pathological stage (P > 0.05; Fig. 3a and c–h).

### Protein expression of CBXs in patients with CRC

**Fig. 4:**
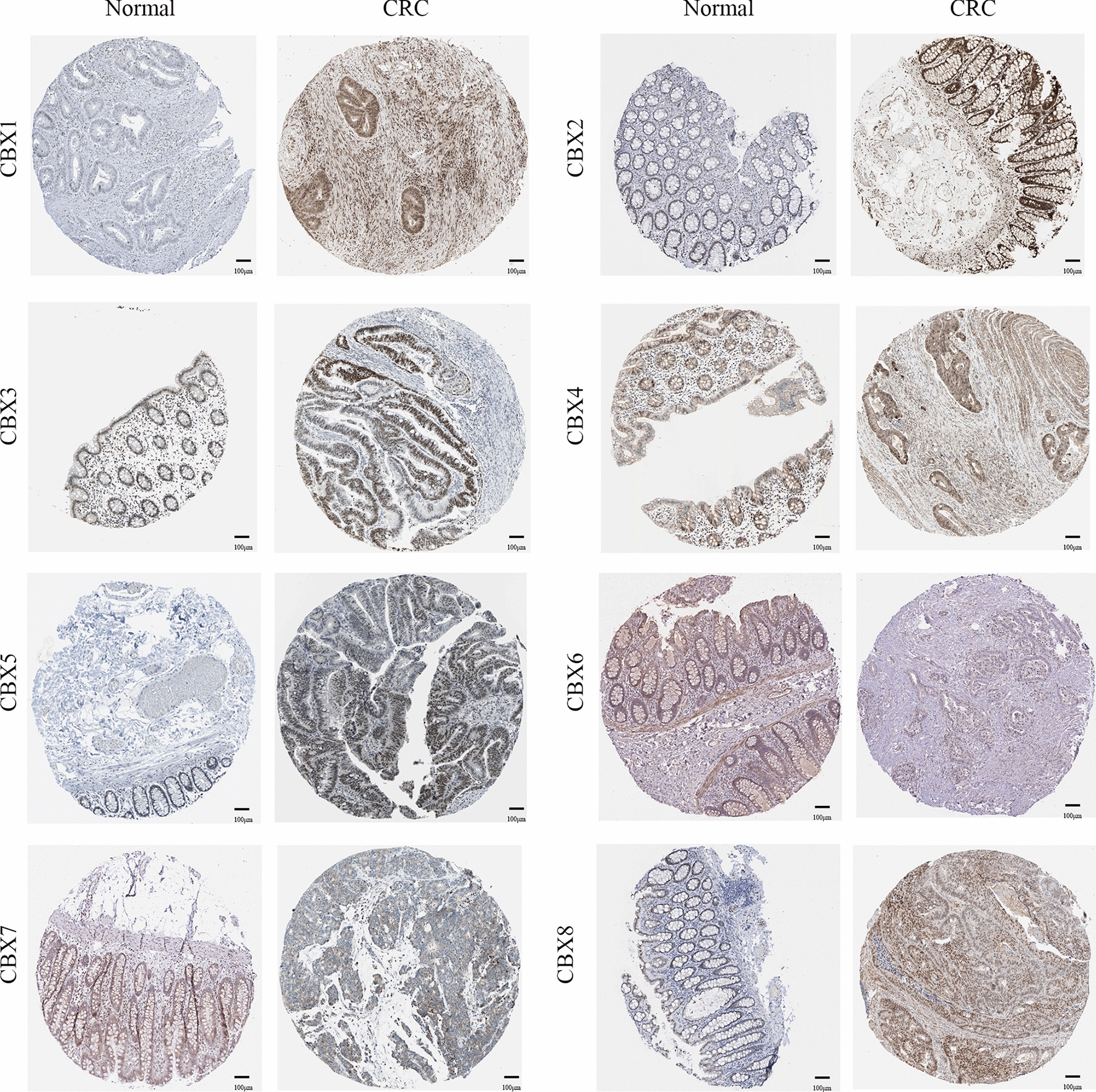
Immunohistochemical analysis of protein expression in CRC and normal tissues (The Human Protein Atlas Database). The brown areas represent positive expression and the blue negative. Scale bar, 100 ;μm. CRC colorectal cancer

To further verify the trend of CBX expression in CRC tissues, the results of IHC analysis of CBXs were obtained from the HPA database (Fig. 4). According to the degree of staining, the protein expression of CBX1-5 and CBX8 in CRC tissues was also higher than that in normal tissues. Conversely, CBX6 and CBX7 were lower in CRC tissues, as compared to normal tissues. These results were consistent with the mRNA expression.

### Association between CBX family expression and prognosis in patients with CRC

**Fig. 5:**
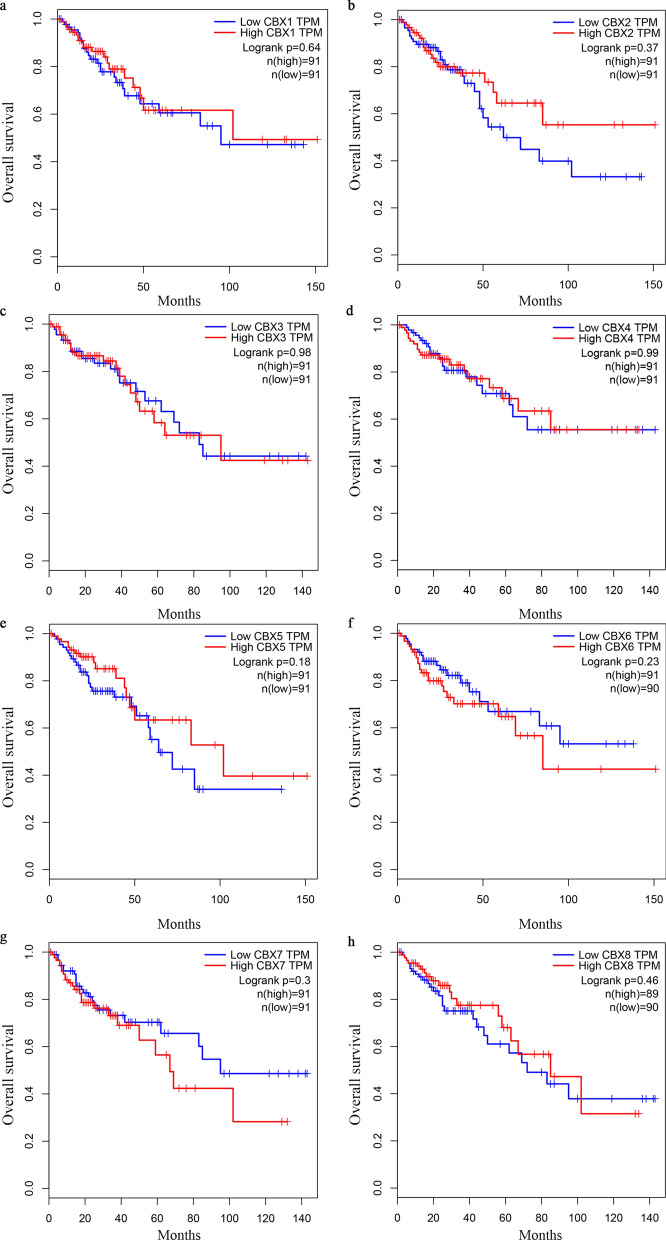
Survival analysis and prognostic values of CBX family in patients with CRC (GEPIA database). **a–h** OS curves of CBX1, CBX2, CBX3, CBX4, CBX5, CBX6, CBX7, and CBX8 in patients with CRC. *OS* overall survival, *HR* hazard ratio, *CRC* colorectal cancer

**Fig.6:**
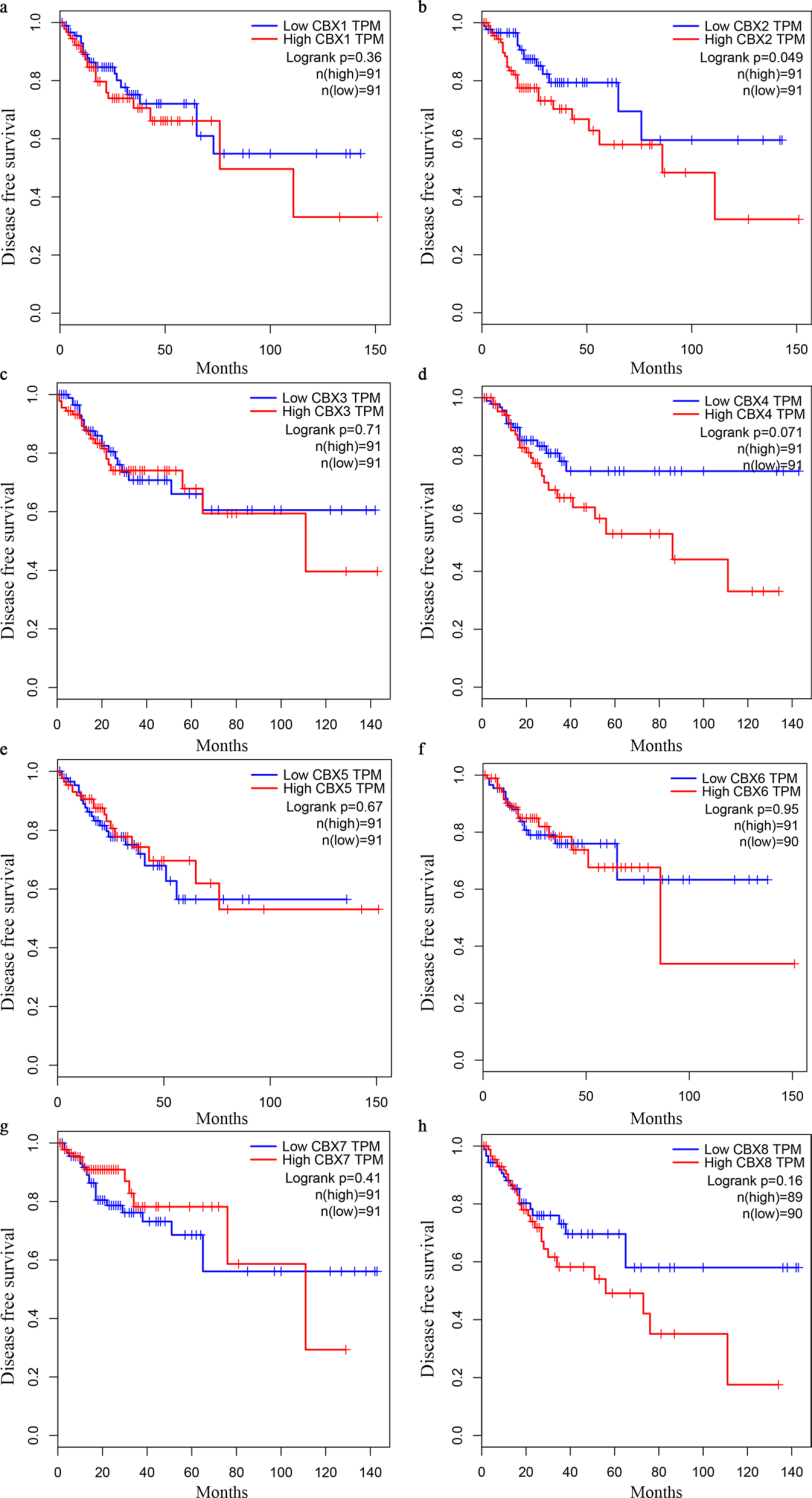
Survival analysis and prognostic values of CBX family in patients with CRC (GEPIA database). **a–h** DFS curves of CBX1, CBX2, CBX3, CBX4, CBX5, CBX6, CBX7, and CBX8 in patients with CRC. *DFS* disease-free survival, *HR* hazard ratio, *CRC* colorectal cancer

Further assessment by GEPIA database analysis of the prognostic effect of the CBX family mRNA expression in CRC revealed that a high CBX2 expression in patients was significantly associated with a worse disease free survival (DFS), as compared with a low CBX2 expression (P = 0.049; Fig. 6b). However, no significant difference (P > 0.05) was observed in the OS and DFS associated with or without mRNA expression alteration in the remaining CBX family members (Figs. 5 and  6a and c–h). A comparison of the above databases indicated that CBX2 might be a potential prognostic target for CRC.

### CBX2 knockdown inhibited CRC cells proliferation and invasion

**Fig. 7:**
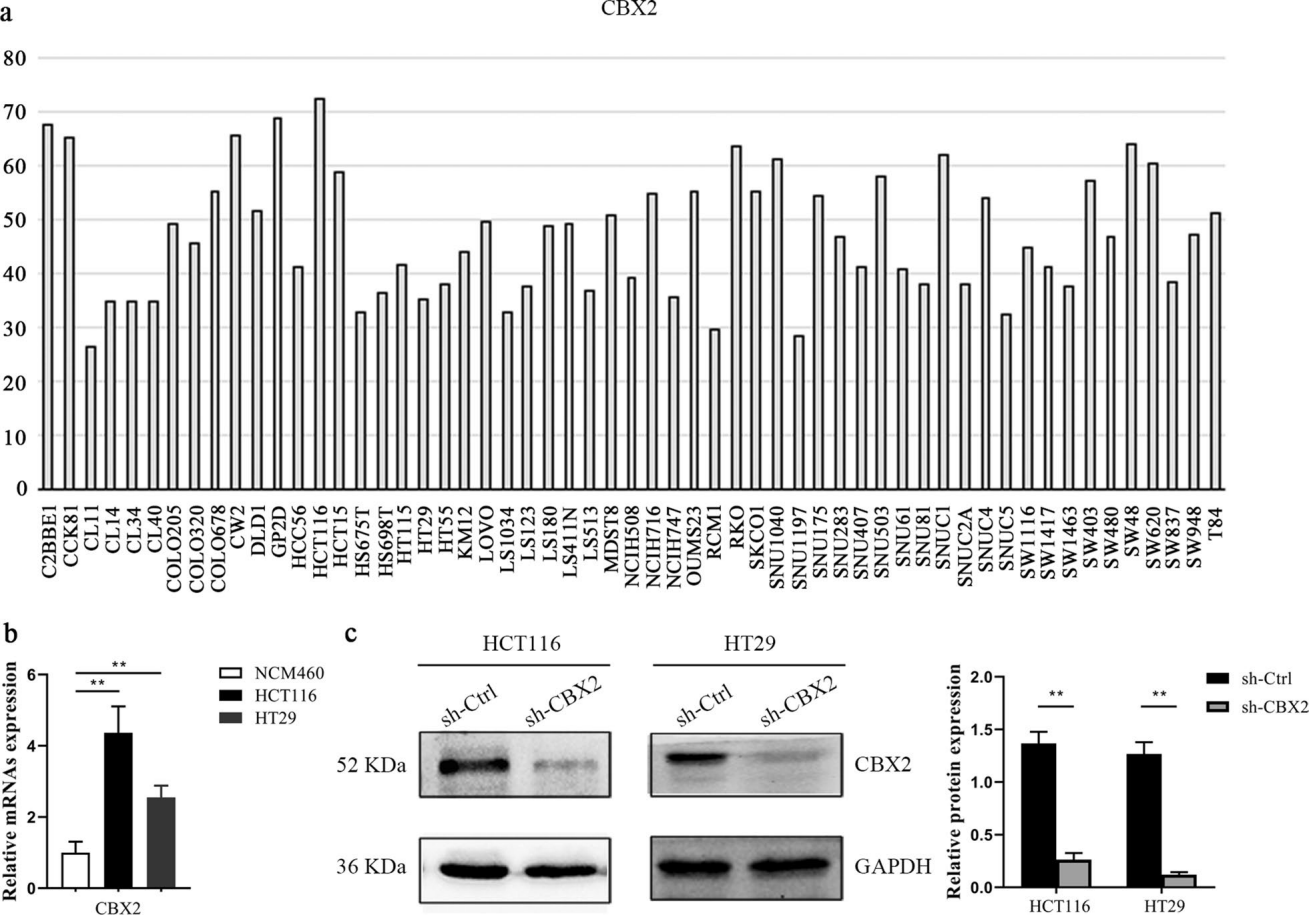
CBX2 is upregulated in CRC cell lines. **a** mRNA expression level of CBX2 in different CRC cell lines, as determined by CCLE analysis. **b** The mRNA expression level of CBX2 in HCT116, HT29, and normal colon mucosal epithelial cell lines was detected by RT-qPCR (n = 3 independent experiments. **P < 0.01). **c** The protein expression level of CBX2 in stably-transduced sh-Ctrl and sh-CBX2 HCT116 and HT29 cell lines was analyzed by western blotting (n = 3 independent experiments. **P < 0.01). *CRC* colorectal cancer

**Fig. 8:**
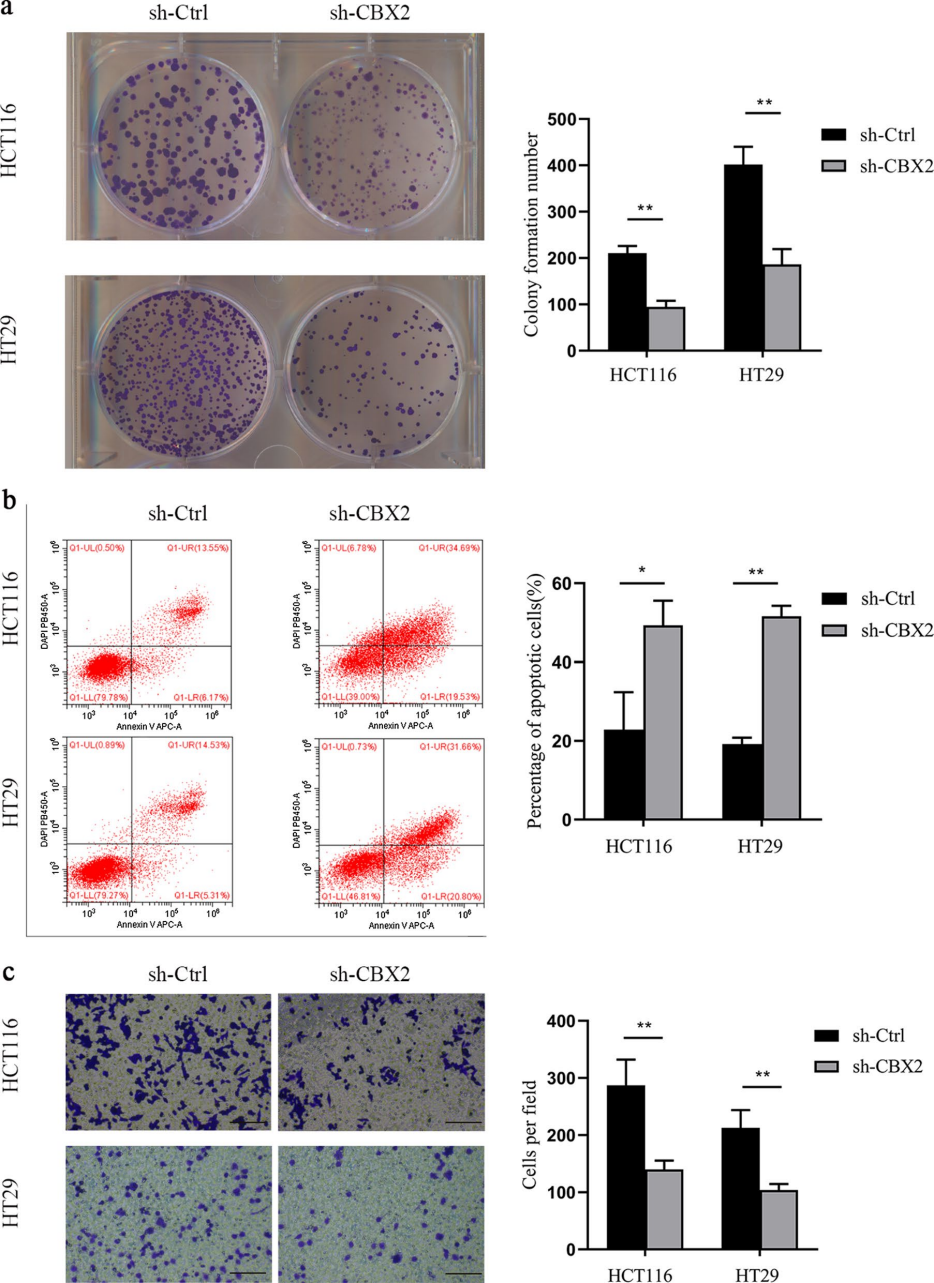
CBX2-knockdown inhibits CRC cell proliferation and invasion. **a** CBX2-knockdown suppressed cell proliferation, as determined by colony formation assays in HCT116 and HT29 cell lines (n = 3 independent experiments *P < 0.01). Scale bar, 100 magnification. **b** Apoptosis ratios of CBX2 in stably-transduced sh-Ctrl and sh-CBX2 HCT116 and HT29 cell lines were detected by flow cytometric analysis (n = 3 independent experiments *P < 0.025, **P < 0.01). **c** Knockdown of CBX2 significantly inhibited cell invasion in HCT116 and HT29 cell lines (n = 3 independent experiments, **P < 0.01)

Through the CCLE database, we found that the expression of CBX2 in HCT116 and HT29 cell lines was significantly higher, compared with other CRC cell lines (Fig. 7a). Subsequently, the results in the CCLE database were verified by RT-qPCR experiment (Fig. 7b). Accumulating evidence has demonstrated that cancer is closely associated with abnormal proliferation [23]. A large number of cases in the GEPIA database have suggested that an elevated CBX2 expression can lead to poor prognosis. We therefore wondered whether CBX2 was involved in maintaining the malignant phenotype of CRC cells. To explore the biological function of CBX2 in the tumorigenesis of CRC, we investigated whether CBX2-knockdown could inhibit cell proliferation. First, as shown in Fig. 7c, the protein expression of CBX2 was significantly inhibited in HCT116 and HT29 cell lines following transfection by western blotting. Next, a colony formation assay indicated that CBX2-knockdown led to a marked reduction of colony numbers in HCT116 and HT29 cell lines (Fig. 8a). Furthermore, flow cytometry, performed to determine whether apoptosis was involved in the CBX2-knockdown-induced inhibition of proliferation, showed apoptotic cells were significantly increased in the CBX2-knockdown group, as compared with the sh-Ctrl group (Fig. 8b). In addition, as compared with the sh-Ctrl group, transwell assays revealed a significant reduction in cell invasion in the CBX2-knockdown group (Fig. 8c).

## Discussion

It has been well-established that the process of CRC initiation can be attributed to cumulative genomic mutations [24, 25]. Mutations of numerous oncogenes and tumor suppressor genes during the multiple-step evolution of CRC could lead to the transformation of tissues from normal epithelial to carcinoma [26]. In addition to gene mutations that lead to the activation of proto-oncogenes or inactivation of tumor suppressor genes, abnormal alterations in epigenome modification also play a key role in the initiation and progression of a variety of cancers, including lung, breast and liver cancer, and CRC [27–30].

Epigenetics, defined as a stable genetic phenotype resulting from changes in chromosomes without changes in DNA sequence. Several studies have indicated that endogenous and exogenous stimulation can reorganize the chromatin structure of cells, resulting in the expression or suppression of abnormal genes, allowing them to obtain the hallmarks of cancer [31, 32]. In addition to the appearance of tumor markers, epigenetic alterations are another pivotal events during the cancer process, including abnormal DNA methylation and histone modifications [33]. Cumulative evidence has shown that chromosomal instability and microsatellite instability are the two main ways that cause CRC genome instability. However, emerging evidence has identified a third type of CRC characterized by DNA hypermethylation, which is defined as having a CpG island methylation phenotype (CIMP) [34]. At present, pathological examination based on the pathological staging and histological characteristics of tumors is still the most accurate method to assess the prognosis of patients with CRC. Several studies suggested that CIMP-positive tumors can lead to a poor overall survival prognosis, and show a close correlation with clinicopathological and molecular characteristics, including tumor location, gender, and whether there are KRAS and BRAF mutations [35]. Moreover, a cohort study of 206 patients with stage III CRC suggested that CIMP-positive is associated with unfavorable prognosis [36]. Futhermore, some researches indicated that poor prognosis of CIMP-positive CRC is due to BRAF mutations [37]. Therefore, the reversibility of epigenetic therapies for these changes has profound implications for the prevention and clinical prognosis of cancer patients [38].

CBX2, also known as CDCA6 or M33, is a crucial component of PcG histone complexes involved in epigenetic controls [39]. The PcG complexes are highly conserved in evolution and contain a variety of enzymes that catalyze histone modification to act as gene suppressors or activators [40]. Increasing evidence has suggested that phenotypic changes caused by histone posttranslational modification dysregulation are one of the pathogenic mechanisms of human carcinogenesis. These events are often described as the biological transformation of cellular molecular hallmarks into a malignant molecular phenotype process [41]. Epigenetic control has been considered a prior response to gene activity caused by changes in chromatin structure, which stems from self-maintenance, post-translational modification of mRNA, and binding between different histones [42]. Hence, further research on the epigenome will greatly improve our understanding of the mechanisms of complex diseases, including cancer. For instance, Chen et al. found that CBX2 was abnormally highly expressed in breast cancer, and an elevation that led to poor prognosis [43]. Clermont et al. reported that CBX2 suppressed cell viability by activatingcaspase-3 enzyme, which caused apoptosis in metastatic prostate cancer cells [44]. Further studies found the knockdown of CBX2 to facilitate the sumoylation activation of SUMO2/3, leading to the occurrence of leukemia [45]. Mechanistically, it was revealed by Mao et al. [16] that CBX2 could activate the Hippo pathway via the downregulation of the Yes-associated protein (YAP) expression, thus regulating the proliferation, apoptosis and DNA repair of hepatocellular carcinoma cells. Moreover, CBX2 was also reported by Han et al. [46] to act as a tumor promoter by binding miRNA let-7a to downregulate the expression of rat sarcoma virus (RAS), resulting in the progression of osteosarcoma. In addition, previous studies have confirmed the correlation between abnormal expression of some members of the CBX family and tumor prognosis. For example, elevated expression of CBX1/2/3/6/8 are associated with poor overall survival (OS) in hepatocellular carcinoma (HCC) patients [47]. Liang et al. found that high expression of CBX1/2/3 are correlatated with unfavorable relapse-free survival (RFS) in breast cancer (BC) patients [48]. Jiang et al. confirmed that CBX4 overexpression is associated with poor prognosis in cell renal cell carcinoma (ccRCC) patients [49].

In the present study, a total of eight CBX family members were evaluated in 20 common human samples and normal control tissues through the Oncomine database. The Oncomine database results showed that most members of CBX were highly expressed in CRC, implying their unique roles in the disease. The present analysis further confirmed this conclusion based on a large-sample TCGA cohort study. Intriguingly, in the survival analysis of CBX family members in patients with CRC, it was found that only a high CBX2 mRNA expression was associated with poor outcome, as compared with patients with a low mRNA expression. These findings suggested that CBX2 may be a potential prognostic target. Further analysis of the CBX2 function found it to be highly expressed in CRC cell lines HCT116 and HT29, as compared to normal colon mucosal epithelial cell line. These results were consistent with the CCLE database. As mentioned above, CBX2 has been proven by numerous researchers to affect cell proliferation by binding to the Ink4a/Arf locus [50]. Consistent with previous observations, the present in vitro experiments indicated that CBX2-knockdown significantly inhibited cell proliferation and invasion in HCT116 and HT29 cell lines. In addition, Daub et al. performed a proteomics study to search for cellular targets in cancer cells, and found that CBX2 was involved in the cell cycle, whose dysregulation could induce apoptosis [51]. Through apoptosis assays, it was confirmed that the downregulation of CBX2 could markedly promote apoptosis in HCT116 and HT29 cell lines.

Inevitably, our research has some limitations. Firstly, we only studied the expression of the CBX family in CRC, while the mutation of the CBX family in CRC has not been further explored. Secondly, we have not further explored the mechanisms of epigenetics, such as signaling pathways. Thirdly, in the apoptosis assay, the cell viability value is relatively low (less than 85%), but we will improve the experimental method in future research work. Even though the underlying molecular mechanism between the abnormal expression of CBX2 and CRC requires more in-depth research, this study clearly confirmed that CBX2 is upregulated in CRC and CBX2-overexpression is significantly associated with poor survival outcomes. In conclusion, the present study suggested that CBX2 could function as an oncogene and serve as a potential prognostic biomarker in CRC.

## Data Availability

The datasets generated and analyzed during the current study are publicly available from the following online databases: CCLE (https://portals.broadinstitute.org/ccle/home); Oncomine database (http://www.oncomine.org); HPA database (https://www.proteinatlas.org/); GEPIA database (http://gepia.cancer-pku.cn/).

## Abbreviations

CBX: Chromobox
RT-qPCR: Real time quantitative polymerase chain reaction
CCLE: Cancer cell line encyclopedia
HPA: Human protein atlas database
GEPIA: Gene expression profiling interactive analysis
TCGA: The Cancer Genome Atlas
YAP: Yes-associated protein
RAS: Rat sarcoma virus
