# Correction to: Expression and prognostic significance of CBX2 in colorectal cancer: database mining for CBX family members in malignancies and vitro analyses

**DOI:** 10.1186/s12935-021-02147-9

**Published:** 2021-08-24

**Authors:** He Zhou, Yongfu Xiong, Zuoliang Liu, Songlin Hou, Tong Zhou

**Affiliations:** 1 The Second Department of Gastrointestinal Surgery, Afliated Hospital of North Sichuan Medical College, 63 Wenhua Road, Nanchong, 637000 Sichuan China; 2 The First Department of Hepatobiliary Surgery, Afliated Hospital of North Sichuan Medical College, Nanchong, 637000 Sichuan China; 3 Institute of Hepatobiliary, Pancreatic and Intestinal Disease, North Sichuan Medical College, Nanchong, 637000 Sichuan China

## Correction to: Cancer Cell Int (2021) 21:402 10.1186/s12935-021-02106-4

Following the publication of the original article [1], we were notified that the name of the second author has been incorrectly spelled as Yong Xiong instead of Yongfu Xiong. The duplicate name for Yongfu Xiong has been removed from the end of the authorship list.

The original article has now been corrected.
